# Sea-anemone toxin ATX-II elicits A-fiber-dependent pain and enhances resurgent and persistent sodium currents in large sensory neurons

**DOI:** 10.1186/1744-8069-8-69

**Published:** 2012-09-15

**Authors:** Alexandra B Klinger, Mirjam Eberhardt, Andrea S Link, Barbara Namer, Lisa K Kutsche, E Theresa Schuy, Ruth Sittl, Tali Hoffmann, Christian Alzheimer, Tobias Huth, Richard W Carr, Angelika Lampert

**Affiliations:** 1 Institute of Physiology and Pathophysiology, Friedrich-Alexander-Universität Erlangen-Nürnberg, Universitätsstraße 17, 91054, Erlangen, Germany; 2 Department of Anesthesiology, Ludwig-Maximilians University, Munich, Germany; 3 Department of Physiological Genomics, Ludwig-Maximilians University, Munich, Germany; 4 Department of Anesthesiology, Medical Faculty Mannheim, Heidelberg University, Mannheim, Germany

**Keywords:** Patch-clamp, Psychophysics, Differential nerve block, Sensory neurons, Itch, Sodium channels, RT-qPCR, *SCN4b*

## Abstract

**Background:**

Gain-of-function mutations of the nociceptive voltage-gated sodium channel Nav1.7 lead to inherited pain syndromes, such as paroxysmal extreme pain disorder (PEPD). One characteristic of these mutations is slowed fast-inactivation kinetics, which may give rise to resurgent sodium currents. It is long known that toxins from *Anemonia sulcata*, such as ATX-II, slow fast inactivation and skin contact for example during diving leads to various symptoms such as pain and itch. Here, we investigated if ATX-II induces resurgent currents in sensory neurons of the dorsal root ganglion (DRGs) and how this may translate into human sensations.

**Results:**

In large A-fiber related DRGs ATX-II (5 nM) enhances persistent and resurgent sodium currents, but failed to do so in small C-fiber linked DRGs when investigated using the whole-cell patch-clamp technique. Resurgent currents are thought to depend on the presence of the sodium channel β4-subunit. Using RT-qPCR experiments, we show that small DRGs express significantly less β4 mRNA than large sensory neurons. With the β4-C-terminus peptide in the pipette solution, it was possible to evoke resurgent currents in small DRGs and in Nav1.7 or Nav1.6 expressing HEK293/N1E115 cells, which were enhanced by the presence of extracellular ATX-II. When injected into the skin of healthy volunteers, ATX-II induces painful and itch-like sensations which were abolished by mechanical nerve block. Increase in superficial blood flow of the skin, measured by Laser doppler imaging is limited to the injection site, so no axon reflex erythema as a correlate for C-fiber activation was detected.

**Conclusion:**

ATX-II enhances persistent and resurgent sodium currents in large diameter DRGs, whereas small DRGs depend on the addition of β4-peptide to the pipette recording solution for ATX-II to affect resurgent currents. Mechanical A-fiber blockade abolishes all ATX-II effects in human skin (e.g. painful and itch-like paraesthesias), suggesting that it mediates its effects mainly via activation of A-fibers.

## Background

The voltage-gated sodium channel subtype Nav1.7 plays a major role in human pain perception: Patients who lack functional Nav1.7 due to loss of function mutations are incapable of feeling pain [1]. Patients carrying mutations that lead to a gain of function of Nav1.7, on the other hand, suffer from inherited pain syndromes, such as the paroxysmal extreme pain disorder (PEPD, [2,3]). In humans nine different subtypes of sodium channels are expressed (Nav1.1 to Nav1.9), and six of them can be found in sensory neurons (the tetrodotoxin sensitive (TTXs) channels Nav1.1, 1.2, 1.3, 1.6 and 1.7, and the TTX resistant (TTXr) channels Nav1.8 and 1.9).

Voltage-gated sodium channels are responsible for action potential (AP) initiation in neurons and propagation along axons [4]. Upon depolarization, sodium channels open rapidly and inactivate within milliseconds, supporting membrane repolarization. On a molecular level, the inactivation gate, which is situated on the linker between the channel’s domains III and IV, swings into the open pore and thereby blocks the permeation pathway for sodium ions [5]. An endogenous blocking particle, most probably the C-terminus of the β4-subunit, may interfere with this process, allowing the induction of resurgent currents [6], that increase neuronal excitability. Resurgent currents are enhanced by conditions that slow fast inactivation, as this increases the possibility for the blocking particle to bind to the open channel. PEPD mutations have a slowed fast inactivation and exhibit resurgent currents when expressed in DRGs [7]. This also holds for HEK293 cells, provided that parts of the β4-subunit are present in the intracellular solution [8,9].

Several toxins are known to interact with the gating properties of voltage-gated sodium channels. ATX-II from the sea anemone *Anemonia sulcata* was shown to slow fast inactivation [10-12], and is therefore likely to induce resurgent currents. When divers get in contact with sea anemone, they report symptoms such as pain and itch. In order to learn more about the potentially painful effects of ATX-II on nociceptive sodium channel gating, we investigated small and large diameter DRGs with the whole-cell patch-clamp method. We can indeed show that ATX-II enhances persistent and resurgent currents in large diameter sensory neurons of the dorsal root ganglia (DRGs), which are thought to be linked to A-fibers of peripheral nerves [13]. Small DRGs on the other hand, which give rise to C-fibers, were not reported to display any endogenous resurgent currents [14] and also application of ATX-II failed to induce them. In order to correlate our findings with human sensations, we injected small amounts of ATX-II intradermally and examined the evoked sensations in healthy human subjects. Our results suggest that ATX-II may selectively activate A-fibers and thereby mediate itch-like sensations and pain.

## Results

### ATX-II increases resurgent and persistent currents in large diameter DRGs

**Figure 1:**
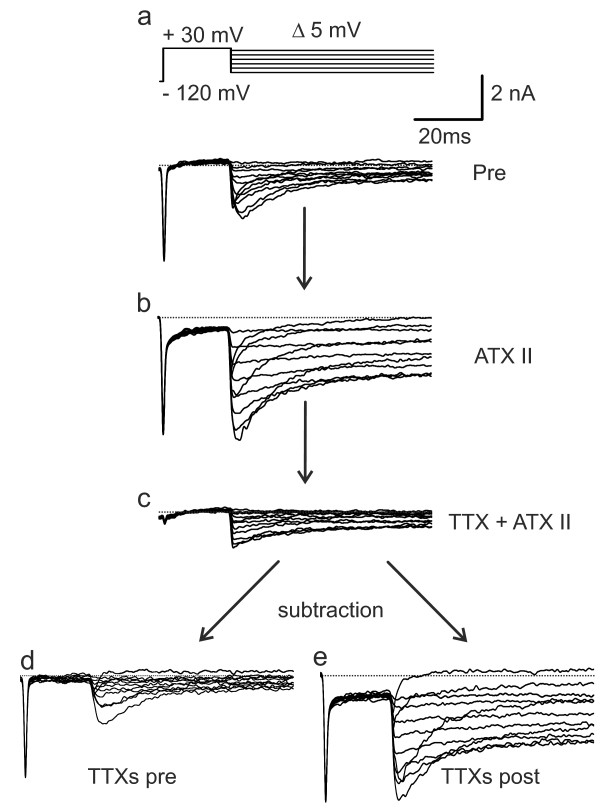
**Example traces for the isolation of ATX-II enhanced TTXs resurgent currents.** Representative recordings from one large diameter DRG neuron using the protocol shown in the upper panel in a. (**a**) Traces recorded under control conditions without any toxin present. (**b**) Traces recorded with 5nM ATX-II in the extracellular recording solution. (**c**) shows recordings in the presence of 5 nM ATX-II and TTX with no resurgent currents present. This trace was subsequently subtracted from the ones shown in (a) and (b), revealing the TTXs resurgent current pre (**d**) and post (**e**) application of ATX-II, respectively. Eight out of eight large DRGs recorded at 22°C showed resurgent currents.

**Figure 2:**
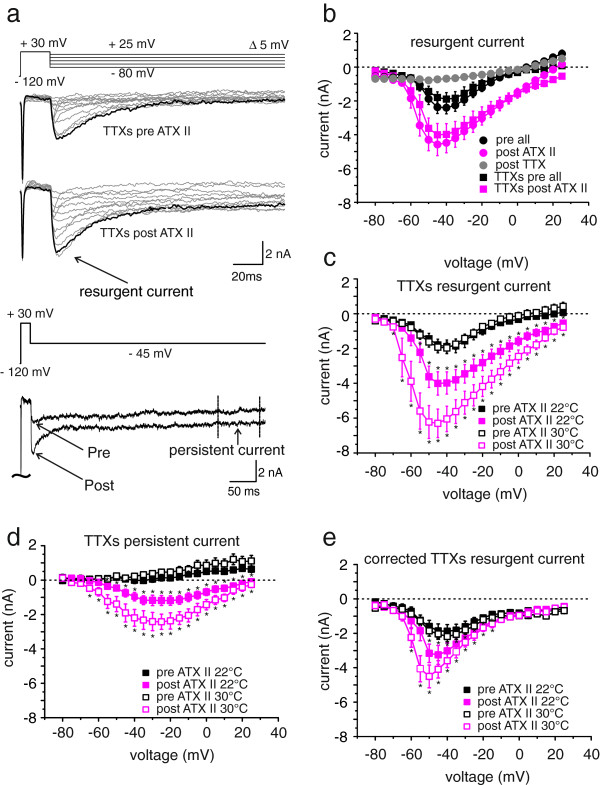
**ATX-II induces resurgent currents in large diameter DRGs.** (**a**) Voltage protocol and representative TTXs resurgent current traces (black traces represent recordings at −45 mV) pre and post application of ATX-II in large diameter DRGs. Lower lane shows an overlay of traces recorded pre and post ATX-II at −45 mV on a longer time scale. Arrows illustrate resurgent current and the region of mean persistent current measurements. (**b**) Peak resurgent current as a function of voltage recorded at 22°C (n = 8). Total current (TTXs and TTXr, black circles), peaked around −40 mV. Absolute total resurgent current (black circles) increased following application of 5 nM ATX-II (pink circles), and is mostly carried by TTXs sodium currents (square symbols). The overall resurgent current was dramatically reduced by application of TTX (grey circles). (**c**) TTXs resurgent current at 22°C (filled squares, n = 8) and 30°C (open squares, n = 14) is increased by application of ATX-II. Data points for 22°C are the same as in (b) and shown for better comparison. (**d**) Mean persistent TTXs current (black squares, determined as shown in (a) lower panel) is increased by ATX-II exposure (pink squares), whereas an increase in temperature has a smaller effect (22°C: filled squares, n = 8; 30°C: open squares, n = 14). (**e**) Corrected TTXs resurgent current amplitudes as a function of voltage. Corrected traces were obtained by subtraction of TTXs persistent current (shown in d) from TTXs peak resurgent current (shown in c) of each trace. At 22°C (filled symbols, n = 8) as well as at 30°C (open symbols, n = 14) corrected resurgent currents are increased by ATX-II application. * p < 0.05, paired-sample T-test.

Large DRGs are known to display resurgent currents [14]. As ATX-II impairs fast inactivation of sodium channels [10,15,16], we set out to test whether it might favor binding of the blocking particle and therefore enhance resurgent currents in DRGs. Upon repolarization following a strong depolarizing pulse (to +30 mV) we evoked resurgent currents in large DRGs that are clearly distinguishable from tail currents by their slower activation and decay kinetics (Figure 1). At the end of the 500 ms repolarizing pulse, a persistent current component was obvious (Figure 2a).

Resurgent currents were clearly increased in large DRGs by addition of 5 nM ATX-II to the bath solution (Figures 1 and 2a and b). As expected for a fast binding toxin like ATX-II, the effect was clearly visible after a few seconds, and we started recordings 1.5 min after toxin application. Although ATX-II most likely increases tail current as well, the slow kinetics of the ATX-II induced current strongly suggests that ATX-II affects resurgent currents. TTX application reduced the total inward current, and resurgent currents were abolished, leaving only a marginal component (Figure 2b, grey circles). This indicates that resurgent currents are mainly mediated by TTXs channel-subtypes. Therefore in the following we isolated the TTXs sodium current by subtracting the TTXr component (Figure 1).

We have previously shown that resurgent currents are modified by the anticancer agent oxaliplatin in a temperature dependent manner [17], and therefore tested the effect of ATX-II on large diameter DRGs at 22°C and 30°C. Native TTXs resurgent or persistent currents were not affected by temperature (Figure 2c and d, black and white symbols). In the presence of ATX-II resurgent and persistent currents tended to be larger at 30°C compared to 22°C (Figure 2c and d, statistically not significant, peak current densities: At 30°C, resurgent 92.2 ± 8.8 pA/pF, persistent 37.4 ± 5.9 pA/pF, n = 14. At 22°C, resurgent 67.6 ± 9.9 pA/pF, persistent 20.4 ± 3.4 pA/pF, n = 8). From Figure 2c the activation of the TTXs resurgent current seems to be shifted to more negative potentials. However, when calculated as relative conductance, this shift is no longer detectable, suggesting that ATX-II solely enhances resurgent currents, and does not alter its voltage-dependence ( Additional file 1: Figure S1).

ATX-II induces a prominent persistent current component (Figure 2d), which may also affect the absolute resurgent current amplitude. In order to evaluate the ATX-II induced resurgent current amplitude in isolation, we subtracted the mean persistent currents measured at the end of the hyperpolarizing pulse from the peak inward resurgent currents (see Figure 2a, lower traces). It is evident, that a large component of the ATX-II effect is due to an increase in persistent currents (Figure 2e compared to Figure 2c). Nonetheless, corrected resurgent currents (Figure 2e) are enhanced by ATX-II at both temperatures tested (corrected resurgent current densities: 22°C pre: 35.5 ± 7.7 pA/pF; 22°C post: 57.0 ± 9.7 pA/pF, n = 8. 30°C pre: 47.6 ± 5.4 pA/pF; 30°C post: 106.9 ± 9.2 pA/pF, n = 14).

Steady-state fast inactivation of TTXs Navs in large DRGs was shifted to more hyperpolarized potentials by application of 5 nM ATX-II (Vhalf pre: -62.5 ± 1.2 mV, post: -66.8 ± 1.2 mV, p < 0.001), as was the voltage dependence of activation (Vhalf pre: -42.6 ± 1.1 mV, post: -45.0 ± 0.9 mV, p < 0.01, Additional file 2: Figure S2a). Surprisingly, a double-exponential fit to current decay did not reveal significant changes when cells were exposed to 5 nM ATX-II ( Additional file 2: Figure S2b and 2c). It may be that the changes remained small and under our detection level, or that the underlying TTXs channel subtypes are affected to a different extent.

### ATX-II is unable to induce resurgent currents in small DRGs

**Figure 3:**
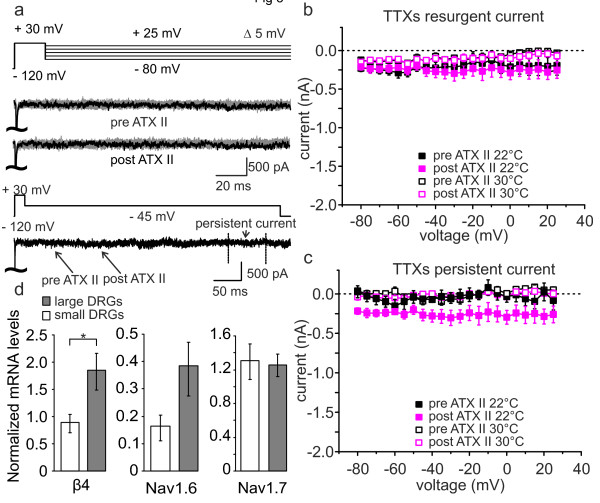
**Small diameter DRGs are unaffected by ATX-II or increased temperature.** (**a**) The same stimulation protocol as used in large diameter DRGs was applied to small DRGs (upper lane). Example traces of TTX-s sodium currents pre and post 5 nM ATX-II application are shown. Lower lane displays an overlay of pre and post ATX-II traces at a voltage of −45 mV. (**b**) TTXs peak current at the time point at which resurgent currents would be expected as a function of voltage in small diameter DRGs. Neither at 22°C (filled symbols, n = 7) nor at 30°C (open symbols, n = 4) a significant ATX-II- induced resurgent current is evident (paired-sample T-test). (**c**) Mean persistent current as a function of voltage pre (black symbols) and post (pink symbols) ATX-II application. In small diameter DRGs there is no significant increase in persistent current detectable due to ATX-II exposure or heating to 30°C (paired-sample T-test). (**d**) Expression levels of β4, Nav1.6 and Nav1.7 mRNA normalized to the mean of two house-keeping genes in FACS-sorted small (white bars) and large (grey bars) DRGs determined by RT-qPCR. In large DRGs ß4 and Nav1.6 expression is twice as high compared to small neurons. On the contrary, Nav1.7 mRNA is evenly expressed in small and large DRGs (n = 3; * p < 0.05).

Up to now, no resurgent currents were described in small diameter DRGs [14], and accordingly, we were unable to detect any significant resurgent or persistent sodium currents in sensory neurons <25 μm (Figure 3a, upper traces, for criteria for resurgent current detection see Methods section). Addition of 5 nM ATX-II at 22°C or 30°C did not induce any detectable resurgent or persistent sodium currents (Figure 3b and c), although application of higher concentrations, such as 25 nM induced a significant persistent current in small DRG neurons ( Additional file 3: Figure S3). In contrast, voltage dependence of activation and steady-state fast inactivation were shifted to more negative potentials by 5 nM ATX-II (Activation: Vhalf pre: -34.9 ± 1.0 mV, post: -40.7 ± 0.8 mV, steady-state fast inactivation: Vhalf pre: -74.7 ± 2.4 mV, post: -80.5 ± 2.0 mV, both p < 0.001, Additional file 2: Figure S2a). A double exponential fit of current decay did not reveal any significant changes when 5 nM ATX-II was applied to small DRGs (see Additional file 2: Figure S2b and c). Our results suggest that either small DRGs possess a set of sodium channels less sensitive to ATX-II, or that they express insufficient amounts of the key components necessary for the generation of resurgent currents. Resurgent currents are thought to rely on the presence of an endogenous blocking particle, which is most likely formed by the β4-subunit, or at least parts of it [9].

Different types of DRGs were suggested to express separate sets of sodium channel α- and β-subunits. In situ hybridization and functional studies suggest that Nav1.6 is predominantly found in large DRGs, whereas Nav1.7 is reported to be functionally responsible for AP propagation in C-fibers [18-20], which are the neurites of small DRGs [13]. β4 was described to be expressed at a higher level in large diameter DRGs than in small [21]. In order to assess the expression of β4, Nav1.6 and Nav1.7 in our preparation, we sorted dissociated DRGs according to their size using FACS (see Methods). The mRNA of size-sorted cells was isolated and we confirmed by RT-qPCR that in our preparation mRNA of the β4-subunit is twice as strongly expressed in large compared to small DRGs (Figure 3d). Nav1.6 mRNA levels were more prevalent in large DRGs compared to small DRGs whereas Nav1.7 mRNA was expressed at comparable levels between the two groups. Thus, the lack of resurgent currents in small DRGs might be due to insufficient amounts of β4-subunits, as there is evidence that the β4-subunit plays an important role in the generation of resurgent currents [9,22].

**Figure 4:**
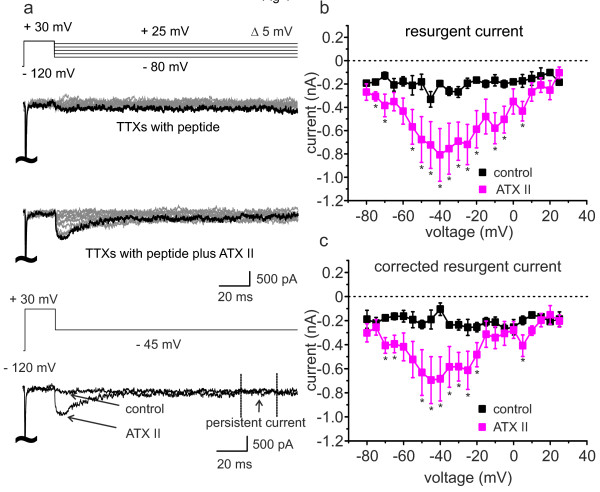
**ATX-II induces resurgent currents in small diameter DRGs with the β4-C-terminus peptide in the pipette solution.** (**a**) Voltage protocol (top panel) and representative current traces of recordings from small diameter DRGs with the β4-peptide added to the pipette solution. Lower lane shows an overlay of recordings from control and ATX-II (5 nM) treated cells at −45 mV. (**b**) TTXs peak current within the first 10 ms after repolarization of control (black symbols, n = 4) and ATX-II exposed (pink symbols, n = 6) small DRGs. Under ATX-II exposure a distinct resurgent current can be recorded, when the β4-peptide is present in the pipette solution. (**c**) The increase of resurgent current is not due to a persistent current component, as subtraction of the persistent current (determined at the end of the hyperpolarizing pulse, see lower panel in a) from peak current reveals a clear corrected TTXs resurgent current component. * p < 0.05, independent-sample T-test.

We added a 14 amino acid peptide of the β4-subunit C-terminus to the pipette solution, which enables the recording of resurgent currents in heterologously expressed sodium channels [8,23,24]. With the β4-peptide added to the pipette solution, we were able to record very small, hardly detectable, resurgent currents in one small diameter DRG out of four (example trace in Figure 4a, upper lane). When 5 nM ATX-II was present in the bath solution, resurgent currents were recorded from four out of six cells. The amount of induced persistent current was small, and correction for it (compare to Figure 2e) still revealed a robust enhancement of resurgent currents in small diameter DRGs by ATX-II when the β4-peptide was added to the pipette solution (Figure 4c).

### Nav1.7 and Nav1.6-mediated resurgent currents are enhanced by extracellular ATX-II

**Figure 5:**
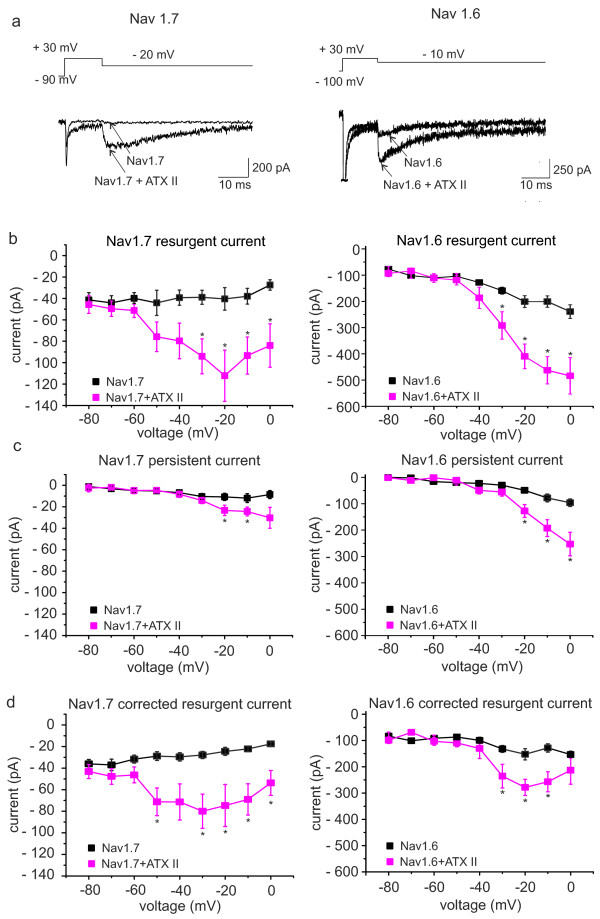
**ATX-II induces resurgent currents in Nav1.7 and Nav1.6 expressing cells when β4-peptide is present in the pipette solution.** (**a**) Voltage protocol (top panel) and representative current recordings with the β4-peptide added to the pipette solution (lower panel): *left*: stable HEK cell line expressing Nav1.7; *right*: Nav1.6 transfected N1E115 cells. An overlay of recordings under control conditions and ATX-II (5 nM) exposure at −20 mV (for Nav1.7) and −10 mV (for Nav1.6) is shown. (**b**) Peak current within the first 10 ms after repolarization of control (black symbols) and ATX-II exposed cells (pink symbols, *left*: Nav1.7, n = 15,17; *right*: Nav1.6, n = 11,11 for control and ATX-II, respectively). Resurgent currents are enhanced by the presence of ATX-II, when the β4-peptide is present in the pipette solution. (**c**) Mean persistent currents at the end of the repolarizing pulse are affected in Nav1.6 (right) and in Nav1.7 (left) by addition of extracellular ATX-II to the bath solution (pink symbols). (**d**) The increase of resurgent current is not due to a persistent current component, as subtraction of the persistent current from peak current reveals a clear corrected resurgent current component in both Nav1.7 (left) and Nav1.6 (right) expressing cells. * p < 0.05, independent-sample T-test for Nav1.7, paired-sample T-test for Nav1.6.

Our results suggest that it is the amount of β4 present in the cell that determines whether ATX-II can increase resurgent currents in DRGs or not. mRNA of Nav1.7 in small DRGs is three to seven times more strongly expressed than Nav1.6, and its expression level does not vary much between large and small DRGs (Figure 3d, [18]). We used a HEK293 cell line stably expressing Nav1.7 and tested for resurgent currents (Figure 5, left column). Up to date no resurgent currents could be evoked when Nav1.7 was transfected either alone or in combination with the β4-subunit, also shown before for Nav1.1 and Nav1.6 [17,23,25]. We were able to record very small resurgent currents in three out of 15 Nav1.7 expressing cells (20.0%) when the β4-peptide was added to the pipette solution. With ATX-II in the recording solution, about half of the cells exhibited resurgent currents (17 out of 32, 53.1%), and their mean resurgent currents were larger than that without ATX-II (Figure 5). ATX-II also increased persistent currents of these cells, but correction for it did not alter the ATX-II effect on Nav1.7 mediated resurgent currents much (Figure 5c, d).

Our RT-qPCR data indicate that Nav1.6 shares its expression pattern between large and small DRGs with β4 and this Nav subtype is linked to resurgent currents in the literature [26-28]. We heterologously expressed Nav1.6 in N1E115 cells and tested for its ATX-II sensitivity with the β4-peptide present in the pipette (Figure 5, right column). In seven out of 17 cells (41.2%) a resurgent current was detectable without ATX-II, which increased significantly when the toxin was applied (twelve cells out of 17, 70.6%). Although the persistent current was enhanced in the presence of ATX-II, too, correction for it still revealed a significant increase of ATX-II induced resurgent currents in Nav1.6 (Figure 5d), which is in accordance with our findings in large DRGs (Figure 2).

**Figure 6:**
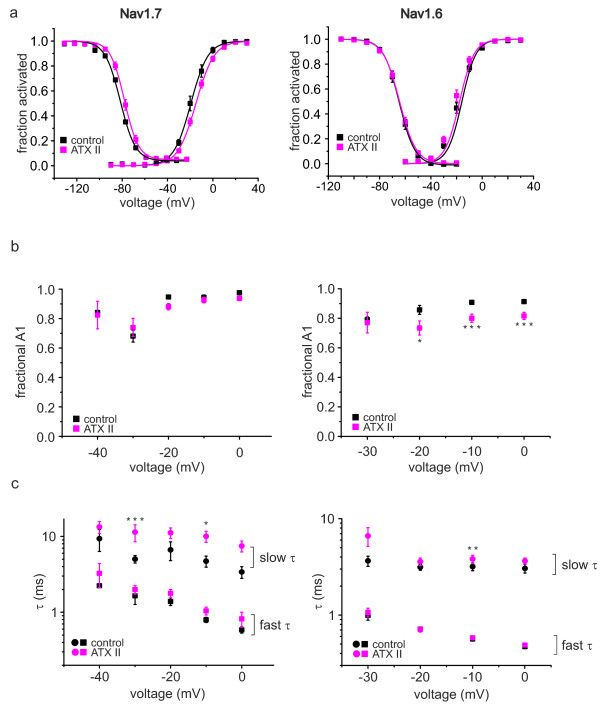
**ATX-II prolongs slow time constant of inactivation of Nav1.7 and Nav1.6.** (**a**) Voltage-dependence of activation and steady-state fast inactivation of heterologously expressed Nav1.7 (left, control n = 8, ATX-II n = 22) and Nav1.6 (right, n = 17), both recorded with the beta4 peptide in the pipette. For results of Boltzmann-fits see text. (b) and (c): Results from a double exponential fit to current decay of traces evoked by the activation protocol from cells expressing Nav1.7 (left, n = 4-22) or Nav1.6 (right, n = 7–17). (**b**) Fractional A1 (A1/(A1 + A2)) is shown as a function of voltage. (**c**) Fast and slow time constants τ of current decay are shown as a function of voltage. In both channel subtypes (Nav1.7 left column, Nav1.6 right column) only slow time constants (circles) are significantly prolonged, whereas fast time constants (squares) are unaffected by ATX-II. Independent-sample T-test for Nav1.7, paired-sample T-test for Nav1.6, * p < 0.05, ** p < 0.01, *** p < 0.001.

Like the resurgent currents, the inactivation kinetics of Nav1.6 seem to be more sensitive to ATX-II than those of Nav1.7 (Figure 6a). A double exponential fit of current decline revealed that in both channels τ 2, the slower time constant is affected by application of 5 nM ATX-II (Figure 6c). Unlike Nav1.7, the relative A1 of Nav1.6 is significantly decreased (Figure 6b), thereby increasing the importance of the slow component in current decay. Slowing the process of inactivation may increase the likelihood of the β4-peptide to bind to the open channel.

In the presence of the β4-peptide, ATX-II shifts the activation of Nav1.6 and Nav1.7 in opposite directions (Vhalf for Nav1.6 pre: -18.2 ± 1.1 mV, post: -20.6 ± 1.2 mV, n = 17; for Nav1.7 control: -19.4 ± 1.6 mV, n = 8, ATX-II: -14.8 ± 1.1 mV, n = 22, p < 0.05, Figure 6a). This opposite effect of ATX-II on Navs suggests that the shift of activation that we see in small DRGs ( Additional file 2: Figure S2a) may be due to ATX-II effects on other Nav subtypes than Nav1.7, or due to the absence of the interacting β4 subunit. The slope of activation remained unchanged (for Nav1.7 control: 6.75 ± 0.41, ATX-II: 7.4 ± 0.29, for Nav1.6 control: 6.3 ± 0.2 ATX-II: 5.9 ± 0.3, both not significantly different).

Steady-state fast inactivation of Nav1.6 and Nav1.7 remained largely unaffected by 5 nM ATX-II, and only the slope of Nav1.6 fast inactivation became marginally less steep (Vhalf for Nav1.7 control: -75.3 ± 2.9 mV, ATX-II: -70.8 ± 1.5 mV, for Nav1.6 control: -64.9 ± 1.2 mV, ATX-II: -64.5 ± 1.2 mV, both not significantly different. Slope for Nav1.7 control: 7.0 ± 0.6, ATX-II: 6.6 ± 0.5, n = 3–8, for Nav1.6 control: 6.3 ± 0.2, ATX-II: 5.9 ± 0.3, n = 17, Figure 6a).

Small DRGs give rise to C-fibers, whereas large DRGs were reported to be linked to A-fibers [13]. As 5 nM ATX-II affected large DRGs and did not induce a resurgent current in small DRGs, we predict that in humans ATX-II should preferentially affect A-fiber-mediated somatosensation, while leaving C-fiber-dependent sensations largely intact. In order to test for ATX-II evoked sensations, we performed a psychophysical trial with healthy volunteers.

### Human sensory response to intradermally injected ATX-II is mediated by A-fibers

**Figure 7:**
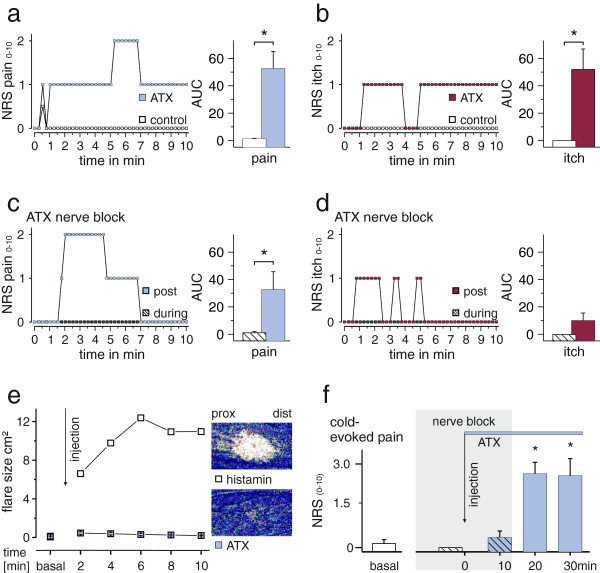
**Intradermal injections of ATX-II induces pain and an itch-like sensations in human volunteers.** (**a**) Pain assessment (on a numerical rating scale, NRS 0–10) as a function of time for a subject following injection of saline solution without (control) or with ATX-II. Under control conditions (white symbols) only a short lasting pain is evoked immediately after the injection in agreement with mechanical irritation, whereas ATX-II injection (blue symbols) leads to distinctly augmented ratings. The right panel shows the mean area under the curve (AUC) for n = 6 volunteers (* p < 0.05). (**b**) Assessment of itch-like sensations on a NRS ranging from 0–10 following injection (sensation rated as 3 is equivalent to desire to scratch) of one representative subject. While under control conditions (white symbols) no itch is induced following injection, ATX-II (red symbols) evoked itch-like sensations, also reflected in a larger AUC (right, * p < 0.05). (**c**) and (**d**) Pain (blue squares and bar) and itch (red squares and bar) ratings as a function of time following compression induced nerve block. After 30–40 min of A-fiber block ATX-II was injected and sensations were rated during 10 min (hatched squares). While no pain and itch is evoked by ATX-II under nerve block conditions, pain (blue symbols) and itch (red symbols) recur fast after removal of the block. AUCs of pain ratings increased significantly after removal of the block (* p < 0.05). (**e**) Area of increased blood flow measured by laser doppler imaging every 2 min following ATX-II injection (blue squares, n = 6) shows hardly any increase in superficial blood flow. For comparison, a typical histamine-induced axon reflex erythema mediated by activation of C-fibers and concomitant CGRP release is shown (white squares). *Right:* Example images taken 4 min after application of substances. (**f**) Assessment of cold-evoked (0°C, 7 s) pain (NRS 0–10) before (white bar) and during nerve block (hatched bars). 10 min after injection of ATX-II cold-evoked pain is rated and the nerve block removed. Cold-evoked pain 20 and 30 min after injection of ATX-II significantly increases following removal of the nerve block (* p < 0.05).

Upon intradermal injection of ATX-II on the forearm, all subjects reported unpleasant and painful prickling or tingling sensations, which were not of burning character. These sensations were described to be different to C-fiber mediated pain as it is for example reported following capsaicin injection. The pain was also described as "pulsating" or “cold snowflakes which are hitting the skin”. In parallel to pain subjects reported itch-like sensations, which were characterized as "if the skin was tickled with a thin hair”, “an insect walked on the skin”, or “liquids dropped on the skin”. Subjects were asked to rate their sensations on a numerical rating scale (NRS, 0–10, with 0 being no pain and 10 the worst imaginable pain) and mean NRS ratings were 1.3 ± 0.1 for both, pain and itch-like sensations in the first 10 min after the injection (Figure 7a and b show representative examples each), and declined within ~10 min after the end of the experiment. A control group was injected with ATX-II-free buffer solution and did not report any itch or pain, apart from a small immediate short lasting pain which was most likely evoked by mechanical irritation due to the injection needle (mean < 0.3 on the NRS, n = 9, 3 male and 6 female).

The AUCs of pain and itch ratings were significantly higher in the ATX-II-injected group compared to the controls (Figure 7a and b, n = 6 each, p < 0.004; p < 0.008, respectively, Mann Whitney U-test). Static or mechanical allodynia was absent (tested by gentle pressure with a cotton swab and stroking with a fine brush 10 min after injection of ATX-II, not shown). NRS pain ratings (0–10) to a 7 s lasting heat stimulus of 47°C were unchanged compared to preinjection conditions (n = 6, not shown).

A typical symptom of C-fiber activation is the axon reflex flare [29]. Following application of ATX-II, laser Doppler scanning detected a small increase in local blood flow due to the injection itself, but there was no widespread axon reflex flare (n = 6, Figure 7e, blue squares). A classical axon reflex erythema evoked by histamine (1% applied by iontophoresis) is shown for comparison (Figure 7e, white squares). In humans, histamine activates the group of mechano-insensitive C-fibers, which release calcitonine gene-related peptide (CGRP) upon activation causing widespread vasodilatation [30] when evoked action potentials are antidromically propagated into branching nerve endings.

Our patch-clamp experiments suggest that ATX-II preferably activates A-fibers (connected to large diameter DRGs), whereas its effect on C-fibers (linked to small DRGs) is small or negligible. For further validation of this hypothesis a mechanical differential nerve block was applied prior to ATX-II injection, which mainly affects A-fibers [31,32]. During differential nerve block, no pain (apart from a brief injection pain) or itch-like sensations were reported, revealing a mean AUC during nerve block of 1.3 ± 0.9 and 0.0 ± 0.0, respectively (hatched squares Figure 7c and d). After removal of the nerve block pain and itch-like sensations immediately arose within seconds and were described as “identical” to those following ATX-II injection without a nerve block by volunteers who had undergone this procedure before. This indicates that mechanical block successfully interfered with ATX-II evoked sensations.

NRS curves for pain and itch were recorded for 10 min following removal of the differential nerve block, and subjects reported spontaneous pain (Figure 7c, blue squares). AUC values for pain ratings post nerve block were significantly higher than during nerve block (n = 6, p < 0.03; Wilcoxon matched pairs test, Figure 7c, bar graph). However, no itch-like sensations were reported by 50% of the volunteers following ATX-II injection after removal of the nerve block, indicative of a faster decline of the itch-like component of ATX-II evoked responses over time compared to pain. This further suggests that ATX-II mediates its effects in humans via A-fibers.

NRS pain ratings in response to a cold stimulus (7 s, 0°C) were used to determine progression of the differential nerve block. Mean ratings before treatment were 0.1 ± 0.1, and cold sensation was lost under nerve compression before injection of ATX-II (Figure 7f, white bar). During the differential nerve block only very little cold evoked pain was recorded after ATX-II injection (Figure 7f, blue hatched bar). Removal of block, however, lead to a significantly increased cold evoked pain rating and discomforting mechanical sensations 20 and 30 min after ATX-II injection compared to basal ratings (both p < 0.002*, ANOVA (p < 0.001), following HSD post hoc test). This phenomenon was limited to the site of ATX-II injection within the area of the differential nerve block. The strongly enhanced cold evoked pain following ATX-II injection most likely depends on A-fiber activation, as it was abolished by differential nerve compression.

## Discussion

Here we show, that ATX-II enhances persistent and resurgent currents in large diameter DRGs (Figure 2), but fails to do so in small DRGs (Figure 3). This is most likely due to a lack of sufficient β4-subunit expression in these small neurons (Figure 3d), which is thought to be essential for the generation of resurgent currents. Addition of the β4-peptide to the pipette solution enabled us to record ATX-II-inducible resurgent currents in small DRGs and HEK cells stably expressing Nav1.7. Large DRGs are linked to A-fibers, and supporting our in vitro findings, injection of small amounts of ATX-II into the skin of human volunteers evoked responses in accordance with A-fiber activation.

Large DRGs exhibit endogenous resurgent currents that can be enhanced by ATX-II. This is not the case for small DRGs, even though small DRGs are known to express ATX-II sensitive sodium channels (such as Nav1.1, Nav1.2 and Nav1.6 [16,18,20,33]). Mice lacking functional Nav1.6 (*med* mice) display reduced resurgent currents [34], and there is evidence, that the interaction of the β4-subunit with sodium channels is a major precondition for resurgent currents to occur (e.g. [35]). Our RT-qPCR data of the FACS sorted DRGs show that large DRGs have higher levels of β4 mRNA than small neurons (Figure 3d and [21,34]). Considering the primer efficiencies, DRGs of both sizes have ~5 times more β4 than Nav1.6 (5 and 4.5 times for small and large DRGs, respectively), which would be ample for a simple 1:1 interaction. In small DRGs Nav1.7 is expressed ~7 times stronger than Nav1.6, making it more likely for the β4-subunit to interact with Nav1.7 than with Nav1.6 in small DRGs. It may be possible that Nav1.7 has a lower affinity for β4, and therefore needs higher concentrations for a successful interaction, which could be provided by addition of β4-peptide to the recording solution (Figures 4 and 5).

Compared to Nav1.7, Nav1.6 shows resurgent currents in a larger population of cells under control condition with the peptide in the pipette (53.1% vs. 26.7%, respectively). Nevertheless it might be hard to compare channels expressed in a cell line and draw conclusions to the extent of its modification in its native environment as for example in DRGs. If the peptide was present, seven out of 17 cells transfected with Nav1.6 show resurgent currents, whereas only three out of 15 cells expressing Nav1.7 showed these currents by themselves. Although the cellular background may play a role, these data suggest that Nav1.6 is more prone to interact with the β4-peptide and to produce resurgent currents. It may be possible that the amount of endogenous β4-subunit in small DRGs is simply not sufficient for a robust interaction with any endogenous TTXs sodium channel expressed in these cells and therefore no resurgent currents can be recorded.

An increase in ATX-II concentration up to 25 nM failed to induce resurgent currents in small DRGs ( Additional file 3: Figure S3), but only enhanced persistent currents. This finding is comparable to that of the application of the wasp venom β-Pompilidotoxin to CA3 neurons [36], which induced tail and persistent, but no resurgent currents unless the β4-peptide was present in the recording pipette. Our data suggest that small DRGs share this characteristic with CA3 neurons.

An enhanced persistent current may very well increase excitability by its own, and indeed application of 1 μM ATX-II to IB4 negative small DRG neurons resulted in a broader AP [37]. Interestingly, ATX-II sensitivity seems to be distinct between the different subsets of small DRG neurons, as IB4 positive small DRGs did not change their AP properties in response to ATX-II application. The toxin concentrations that we used in our study induced a shift of TTXs current activation and steady-state fast inactivation ( Additional file 2: Figure S2), but failed to have effects on persistent or resurgent currents (Figure 3). Therefore, it is likely that the toxin concentrations used in our experiments remained below threshold for an effect on persistent or resurgent currents in small DRGs, and that in small DRGs persistent and resurgent currents are less sensitive to small amounts of ATX-II than activation and steady-state fast inactivation.

Surprisingly, mRNA for Nav1.7 is expressed in large and small DRGs at comparable levels (Figure 3d, [18]). Patients with the inherited pain syndromes erythromelalgia and PEPD, both due to mutations in Nav1.7, describe their pain mainly as burning - a typical C-fiber associated characteristic. As Nav1.7 seems to be evenly distributed between large and small DRGs, it is likely that associated proteins, such as the β4-subunit, or cell-background specific channel modifications, may play a major role in the generation of Nav1.7 related pain. The discrepancy may also be due to species differences: We investigated DRGs from mice, but erythromelalgia and PEPD manifest themselves in humans. Knowledge about the developmental regulation of β4 expression is still lacking. PEPD mutations of Nav1.7 were shown to induce resurgent currents by slowing fast inactivation [7,8]. We show in this study, however, that even in the presence of ATX-II no resurgent current could be evoked in small DRGs or Nav1.7 expressed in HEK293 cells unless the β4-peptide is present. Patients with PEPD are severely affected during their first year of life but symptoms become somewhat milder later in life. It remains speculative if this might be due to a down regulation of β4 with age, for which data are currently not available.

ATX-II alone was unable to induce resurgent currents in heterologously transfected Nav1.7 or Nav1.6, which was reported before [38]. Up to now, only one toxin, the β-scorpion peptide Cn2, was able to induce resurgent currents in a heterologous expression system without the addition of an intracellular peptide in Nav1.6 [38]. These currents were enhanced by ATX-II. This finding suggests that sodium channels need some kind of modification, like treatment with Cn2 or potentially a disease causing mutation, such as PEPD, in order to be capable to produce resurgent currents.

In the 70s and early 80s of the last century, it was observed that ATX-II broadens the AP of crustea peripheral axons, and not much later it became evident that this was due to a slowing of inactivation properties of voltage-gated sodium channels [10,15,39,40]. When applied to large DRGs in high concentrations (10 μM), it significantly slowed τ_h_ of Nav current inactivation [41]. Here, we used concentrations that are an order of magnitude lower and although we used recombinant ATX-II (purity >98%), which may be more specific in its effects than purified ATX-II, the effect on the peak current decay time constants was no longer detectable ( Additional file 2: Figure S2 b,c), but nevertheless resurgent currents were altered significantly. Also, we observed shifts in activation and steady-state fast inactivation. This suggests that even though the effects on inactivation kinetics were small, the concentration of ATX was high enough to enhance resurgent currents, which in large DRGs seem very sensitive to even small channel alterations. Higher concentrations of ATX-II evoked resurgent currents in large DRGs, which were too large to be properly recorded under our conditions. This suggests that 5 nM ATX-II is a concentration at which effects may be evoked in large, but not in small DRGs, thereby allowing a separation of the two groups.

Molecular studies discriminated between sodium channel subtypes in heterologous expression systems for their sensitivity to ATX-II, showing that mainly Nav1.1, Nav1.2 and Nav1.6 are modified at very low concentrations [16,33,38]. These three channels can be found in large and small DRGs [18,20] and in large, but not small DRGs their modification by ATX-II enhances resurgent currents.

ATX-II slows inactivation kinetics for both channels investigated in this study: Nav1.6, which is supposed to be the main carrier of the AP in large DRGs and Nav1.7, which is suggested to play this role in small DRGs [19]. The slower fast inactivation occurs the higher the probability is for a potential open channel blocker to interact with the pore. Consequently, in the presence of the β4-peptide, we were able to significantly increase resurgent currents in both channel subtypes (Figure 5). These experiments suggest that two requirements are necessary for ATX-II to increase resurgent currents: I) an ATX-II sensitive sodium channel (such as Nav1.7 or Nav1.6, but also other subtypes, [16,33,38]) and II) the availability of an intracellular open channel blocker. Both seem to be present in large DRGs, whereas our experiments suggest that small DRGs are missing at least one of the components. As the TTXs sodium channels that were shown to be expressed in small DRGs are almost all sensitive to ATX-II (Nav1.1, 2, 6 and 7, [16,18,20], Figures 5 and 6), even at the low concentrations that we used here, it is quite likely that small DRGs are lacking sufficient open channel blocker. Accordingly, when we added the β4-peptide to the intracellular recording solution, resurgent currents were present in small DRGs (Figure 4).

Recently we showed that the anticancer agent oxaliplatin selectively enhances persistent and resurgent currents in large diameter DRGs at low temperatures [17]. Oxaliplatin is highly effective but unfortunately has dose limiting side-effects: acute and chronic neuropathies, which are both characterized by cold evoked very unpleasant dysaesthesias. These sensations are similar to those experienced by the subjects of the present study following intradermal ATX-II injection. Besides an increased cold perception 20 and 30 min after ATX-II injection a pricking sensation outlasting the cold stimulus was observed (see Figure 6f), indicating ongoing activity in the affected nerve fibers.

ATX-II did not display a marked temperature dependence in its enhancement of resurgent or persistent currents, which is in contrast to oxaliplatin (Figure 2 and [17]). Low temperatures induce a general increase in membrane resistance, which would then allow a small depolarizing current, like resurgent currents, to be potentially sufficient to initiate a following AP. This may explain why the subjects report cold enhanced sensations after ATX-II injection (Figure 7f); even though resurgent currents were not significantly different at 22°C compared to 30°C (Figure 2).

Oxaliplatin failed to affect C-fibers or small diameter DRGs, suggesting that this drug, too, relies on the presence of the β4-subunit for its effects, or displays some degree of subtype specificity. The presence or absence of the endogenous blocking particle might thus render certain types of neurons susceptible for activation by toxins or drugs which act via voltage-gated sodium channels. Thus, focusing only on specific sodium channel subtypes for the treatment e.g. of pain might not be reaching far enough, but the channels functions in their cellular context need to be considered.

Pain sensations *in vivo* may be transmitted via two main types of fibers: the myelinated fast conducting A-fibers, which are connected to large DRGs, and the slower and unmyelinated C-fibers which are linked to small diameter DRGs [13]. We have shown that low concentrations of ATX-II activate large DRGs suggesting that A-fibers convey the ATX-II evoked sensations of the volunteers. Accordingly, mechanical nerve fiber block abolished the ATX-II evoked sensations. Most likely Aδ-fibers mediate the ATX-II induced pain symptoms but may also participate in the itch-like sensations. Recently it was shown that the effects of intradermal insertion of spicules from the pods of a cowhage plant induce intense itching. Aδ-fibers seem to be involved in transmitting this itch [42]. In some subjects, these sensations were markedly reduced by mechanical nerve block. Intradermal injection of ATX-II, on the other hand, evoked itch-like sensations, that were not described as common itch, but unpleasant sensation, for which there is no adequate word. “Tingling” was used by some subjects, which maybe an overlapping sensation induced by ATX-II and cowhage spicules. Apart from Aδ-fibers it is likely that Aß-fibers are activated by ATX-II since sensations of mechanical character were described, such as "like punctured by many thin needles". Possibly, the uncommon simultaneous activation of Aδ- and Aβ-fibers may generate the unique ATX-II mediated perception.

In our *in vivo* experiments the A-fiber block may induce paraesthesias itself, both during and upon recovering from the block. However no paraesthesias were described prior to and after ATX-II injection. Nonetheless, the reported sensations after ATX-II injections and relief of block were comparable to those described when no prior block was applied. The fact that cold evoked pain, which strongly increased after removal of the block, was closely restricted to the injection site (Figure 7f) argues against a general block-induced fiber irritation. Nevertheless, an irritation of the fibers cannot be ruled out, but we estimate it to be rather small in comparison to the effects evoked by ATX-II alone.

Intradermal injection of ATX-II did not evoke a large axon reflex flare (Figure 7e), arguing against a major activation of mechano-insensitive C-fibers [29]. Nevertheless, mechano-sensitive C-fibers may mediate itch without the induction of a major axon reflex flare [43,44]. Subjects tested with cowhage spicules, which activate only mechano-sensitive C-fibers vigorously and Aδ-fibers, report quite different sensations than those injected with ATX-II (burning pain, severe itch, which was also clearly labeled as such). As mechano-sensitive C-fibers are also resistant to differential nerve block and should therefore have transmitted the ATX-II-mediated sensations, we assume that mechano-sensitive C-fibers are not a major target of ATX-II in humans.

## Conclusions

ATX-II enhances persistent and resurgent sodium currents in large diameter DRGs, whereas small DRGs depend on the addition of β4-peptide to the pipette recording solution for ATX-II to affect resurgent currents. Mechanical A-fiber blockade abolishes all ATX-II effects in human skin (e.g. painful and itch-like sensations), suggesting that it mediates its effects mainly via activation of large diameter A-fibers.

We thus established low concentrations of ATX-II as a tool for A-fiber activation, which induces pain and itch-like symptoms in humans. We show that activation mainly of A-fibers can be crucial for unpleasant pain and itch-like sensations, which are not extremely painful but could be distressing and reducing quality of life. We also showed for the first time, that this toxin is able to enhance resurgent currents of large DRGs, and of small DRGs or heterologously expressed Nav1.7, when the β4-peptide is present.

## Methods

### Animals and cell culture

Adult C3HeB/FeJ mice were anaesthetized with halothane (Sigma- Aldrich GmbH, Steinheim, Germany) and decapitated in accordance with ethical guidelines established by German animal protection law, approved by the animal protection committee of Regierung Mittelfranken, Germany. After decapitation, the backbone was removed rapidly and bisected longitudinally in ice-cold PBS (PAA Laboratories GmBH, Pasching, Austria). DRGs were removed, desheated, cleaned and enzymatically dissociated in Dulbecco’s modified Eagle's Medium (DMEM, Gibco-Life technologies, New York, USA) supplemented with collagenase/protease for 45 min at 37°C. Cells were mechanically triturated with small diameter pasteur pipettes in TNB medium (Biochrom AG, Berlin, Germany). Poly-D-Lysin (Sigma- Aldrich GmbH, Munich, Germany) coated cover slips were loaded with the cell suspension and after cells settled for 5–10 min TNB medium was added. DRGs were incubated at 37°C and 5% CO_2_ until experiments were performed at the following day. A stable HEK293 cell line expressing hNav1.7 was cultured under same conditions as DRGs in DMEM containing 500 mg/l G418 (Sigma-Aldrich GmbH). N1E115 neuroblastoma cells were maintained in DMEM medium (Gibco-Life technologies) including 4.5 g/l Glucose, 10% fetal bovine serum (Biochrom AG) and 1% Penicillin/Streptomycin (PAA Laboratories GmbH). One day before transfection N1E115 cells were plated on 3.5 cm dishes (Falcon Corning, IBD) and transfected with Nanofectin (PAA Laboratories GmbH) according to the manufacturers protocol using 1 μg mNav1.6R (TTX resistant [45,46], in pCDNA3 and 0.5 μg EGFP-C1 (Clontech Laboratories, Inc., Mountain View, USA) in order to detect transfected cells via green fluorescence. Cells were recorded 2 to 3 days after transfection.

### Whole-cell patch-clamp recordings and electrophysiology

Whole-cell voltage clamp recordings were performed using glass electrodes with tip resistances of 1.5- 2.0 MΩ, manufactured with a DMZ puller (Zeitz Instruments GmbH, Martinsried, Germany) and filled with an internal solution comprising for DRGs (in mM): 140 CsF, 10 NaCl, 10 HEPES, 1 EGTA and 5 TEA-Cl (adjusted to pH 7.38 with CsOH), for HEK cells (in mM): 135 CsCl, 5 NaCl, 2 MgCl_2_ATP, 10 HEPES and 5 EGTA (adjusted to pH 7.2 with CsOH) and for N1E115 cells (in mM): 125 CsCl, 5 NaCl, 2 Mg_2_ATP, 10 HEPES and 5 EGTA (adjusted to pH 7.2 with CsOH). For recordings of activation and steady-state fast inactivation of DRGs, an internal solution with reduced sodium chloride (2 mM NaCl) was used. The external bathing solution contained for DRGs (in mM): 140 NaCl, 3 KCl, 1 MgCl_2_, 1 CaCl_2_, 10 HEPES, 5 Glucose, 20 TEA-Cl, 0.1 CdCl_2_ (adjusted to pH 7.38 with NaOH), except for recordings of activation and steady-state fast inactivation, where NaCl was reduced to 10 mM, substituted with CholinCl, for HEK cells (in mM): 150 NaCl, 3 KCl, 2 MgCl_2_, 1.6 CaCl_2_, 0.4 CdCl_2,_ 10 HEPES, 10 Glucose, 10 TEA-Cl (pH = 7.4, adjusted with NaOH) and for N1E115 cells (in mM): 145 NaCl, 4 KCl, 2 MgCl_2_, 2 CaCl_2_, 10 HEPES, 10 Glucose, 10 TEA (adjusted to pH 7.4 with NaOH).

DRGs were classified according to their cell size as large (> 35 μm) or small (< 25 μm). Size was measured as the maximal diameter of the cells on a digital image taken with an EC3 Camera (Leica, Wetzlar, Germany) connected to a Leica- DCMI3000B microscope with a 64 x objective. Recordings were performed at either room temperature (22 ± 1°C) or 30 ± 1°C, controlled by an inline solution heater (Warner Instruments, Hamden, USA) with a feedback thermocouple positioned less than 800 μm from the recorded cell. A HEKA EPC-10USB (Lambrecht, Germany) was used for signal recording, low-pass (10 kHz) filtering and digitizing (100 kHz).

For substance application the tip of a perfusion system was positioned within 800 μm of the recorded DRG neuron. Pipette potential was zeroed prior to seal formation and capacitive transients were compensated using C-fast for pipette-capacity correction and subsequently C-slow for cell-capacity compensation (PatchMaster, HEKA, Lambrecht, Germany), the series resistance was compensated by about 50% leaving a mean resistance of 3.8 ± 0.4 MΩ. The absolute peak current (pA) was divided by the capacitance as determined by C-slow compensation to obtain current densities. Leak pulses were applied following the test pulse, and mean leak current was subtracted digitally online corresponding to the P/4 test pulse procedure.

To measure resurgent currents in DRGs an initial 20 ms voltage step from a holding potential of −120 mV to +30 mV was applied followed by 500 ms steps from −80 mV to +25 mV (steps of 5 mV). In HEK293 cells a 12.5 ms depolarizing voltage step from −90 mV to + 30 mV preceded 50 ms steps from −80 mV to +20 mV (steps of 10 mV). In N1E115 cells a 12.5 ms depolarizing voltage step from −100 mV to +30 mV preceded 150 ms steps from −90 mV to 0 mV (steps of 10 mV). The resurgent current quantity was measured by detecting the peak inward current following the pre-pulse. Persistent current was determined as the mean current between 400 ms and 515 ms of the voltage protocol. To show resurgent current in isolation, not affected by the persistent current, the latter was subtracted from the peak inward resurgent current to obtain the isolated resurgent current.

Persistent currents give rise to tail currents upon repolarization. Special care was taken in order not to confound the slower activating and decaying resurgent currents with tail currents. Especially its different voltage dependence and kinetics allowed us to clearly discriminate between the two. Current trajectories were only accepted as resurgent currents, if they clearly showed slowly rising and falling kinetics. In small diameter DRGs only tail currents were visible under control conditions. By choosing the relevant area for analysis, the peak inward resurgent currents could reliably be distinguished from tail currents, although a small contamination cannot be excluded.

By subtracting the TTXr component (Figure 1) we eliminated all TTXr currents including potassium and calcium currents, which may also have tail currents. In accordance with the literature, we were unable to detect any TTXr resurgent currents ([14,17]).

Protocols were recorded while the bath perfusion was constantly running, and repeated with ATX-II and TTX in the external solution. TTXs components were isolated by subtraction using Igor Pro software (Wavemetrics, Lake Oswego, USA).

For recordings of DRGs and HEK293 cells with β4-peptide in the internal pipette solution, cover slips were incubated either in bath solution (control) or in bath solution containing 5 nM rATX-II for 7 to 10 min before begin of the protocol.

N1E115 cells intrinsically express TTX-sensitive Nav1.1 and Nav1.2 (Benzinger et al., 1997), which were suppressed in our experiments by adding 1 μM TTX (Biotrend, Wangen/Zurich, Switzerland) to the bath solution. Cells were mounted on the stage of Axiovert 40 fluorescense microscope (Zeiss, Jena, Germany). TTX and ATX- II were applied using a gravity driven Y-filament. Current signals were recorded in whole-cell voltage-clamp mode at room temperature (22 ± 1°C). Recordings were sampled at 20 kHz (low-pass filter 6 kHz) using an Multiclamp 700B amplifier in conjunction with a Digidata 1322A interface and pClamp10 software (all from Molecular Devices, Sunnyvale, USA). Electrodes were made as stated above. Access resistance in whole-cell configuration was <4 MΩ before series resistance compensation (≥75%).

### FACS sorting and RT-qPCR

For each experiment 20–25 DRGs from two adult mice each were pooled, filtered using a 100 μm cell strainer (BD Falcon) and incubated for at least 30 min on ice with both the nuclei labeling agent Hoechst 33258 (Invitrogen) and the avital marker 7AAD (Invitrogen, labels all dead cells). Using a BD-FACSAria-II cell sorter (Special Order System, BD Biosciences, Core Unit Cell Sorting and Immunomonitoring Erlangen), neurons were sorted for viability and size via their fluorescence and the forward-side-scatter plot. In order to correlate the categories retrieved from the forward-side-scatter plots with the actual cell size, sorted DRGs were cultured for 2–3 h and the mean size of cells in each group was determined via digital images taken with an EC3 Camera (Leica, Wetzlar, Germany) connected to a Leica- DCMI3000B microscope. Cells survived sorting well and had a healthy appearance still several hours after FACS sorting. Cells categorized as small had diameters ranging from 13 to 27 μm (the majority around 18 μm), whereas those categorized as large ranged from 27 μm to 43 μm (the majority around 35 μm). Overall DRGs appeared slightly smaller after FACS sorting compared to those cultivated for 24 h, which might also be due to better attachment of the cells to the cover slip in the latter case.

DRGs for each size-category were collected in PBS, centrifuged and dissolved in Qiazol (Qiagen, Hilden, Germany) for RNA isolation according to manufacturer’s protocol. Potentially remaining DNA was digested with RQ1 RNase-free DNase (Promega, Mannheim, Germany). RNA concentration and purity was assessed with the Nanodrop 2000c spectrophotometer (Peqlab, Erlangen, Germany). RNA was subsequently reverse transcribed with random hexamer primers using the cDNA synthesis kit from Peqlab (Erlangen, Germany). Approximately 5 ng of cDNA was added to the 2x SensiMix SYBR No-ROX Kit (Peqlab, Erlangen, Germany) and mixed with 600 nM of target primer for detection of either ß4-subunit (Scn4b), Nav1.6 (Scn8a) or Nav1.7 (Scn9a) cDNA. To avoid amplification of genomic DNA, exon-spanning primers were designed using Primer3Plus and analyzed by IDT OligoAnalyzer 3.1. BLAST search was applied to ensure specificity. Primer characteristics are shown in Table 1. Reactions were performed in duplicates and carried out using an Eppendorf realplex4 cycler (Eppendorf, Hamburg, Germany). An initial denaturation step at 95°C for 10 min was followed by 40 cycles of denaturation at 95°C for 15 s, annealing at 60°C for 30 s and extension at 72°C for 20 s. The generation of specific PCR products was confirmed by melting curve analysis and gel electrophoresis. Differences in expression levels of target genes in small and large neurons were determined using the ΔΔCq method. HPRT (hypoxanthine-guanine phosphoribosyltransferase) and Rpl13a (ribosomal protein L13a) were used as reference genes to generate a mean value for relative quantification of target gene expression.

**Table 1:** Primer pairs for real-time qPCR

**Genes**	**Accession number**	**Target sites**	**Product size (bp)**	**Efficiency**
Scn8a (Nav1.6)	[NCBI:NM_001077499]		153	93%
for: 5’-CGTACTATTTGACGCAGAAAACTT-3’		Exon 2- 3		
rev: 5’-TCATGCTGAAGACTGAATGTATCA-3’		Exon 3- 4		
Scn9a (Nav1.7)	[NCBI:NM_018852.2]		142	95%
for: 5’-CAGCAAAGAGAGACGGAACC-3’		Exon 18- 19		
rev: 5’-TCTCCTCACACAGCCATCTG-3’		Exon 19- 20		
Scn4b (β4)	[NCBI:NM_001013390]		116	96%
for: 5’-CGAAACATCCAGGATTCTCAT-3’		Exon 2-3		
rev: 5’-TCGTCTTCTCCTTGGTGGA-3’		Exon 3		
Rpl13a	[NCBI:NM_009438.5]		59	99%
for: 5’-GCCTACCAGAAAGTTTGCTTACC-3’		Exon 6-7		
rev: 5’-GGTACTTCCACCCGACCTC-3’		Exon 7-8		
HPRT	[NCBI:NM_013556]		132	94%
for: 5’-TGGAAAGAATGTCTTGATTGTTG-3’		Exon 4-5		
rev: 5’-CGAGAGGTCCTTTTCACCAG-3’		Exon 7		

### Psychophysics

Informed written consent was obtained from all subjects (n = 9; 3 male and 6 female) in accordance with the requirements of the Declaration of Helsinki and experimental procedures for this study on human volunteers were passed by the ethics committee of the University of Erlangen. 70 μl of a sterile control solution (buffer) or solution containing ATX-II (100 nM) were intradermally injected at the volar forearm or to the innervation territory of the superficial radial nerve at the back of the hand of the volunteer. Pain and itch-like sensations were assessed on a numerical rating scale (NRS), ranging from 0 (no pain/itch sensation) to 10 (worst imaginable pain/itch) in 15 s intervals for a period of 10 min. For thermal stimulation metal rods of 1 cm^2^ in diameter were placed in a preheated water bath at 47°C or ice water. Numerical ratings to a 7 s lasting heat stimulus of 47°C and to a 7 s lasting cold stimulus of 0°C were compared before and after application of substances. The skin was tested for static and dynamic mechanical allodynia by applying gentle pressure with a cotton swab and stroking with a fine brush. Laser-Doppler imaging (LDI, Moore, London, UK) was used to record changes in superficial skin blood flow. Two baseline scans of 0.5 mm spatial resolution on a 10.8 cm x 7.0 cm area were taken, followed by scans every 2.0 min starting right after the injections. The skin blood flow was analyzed with MLDImg 3.08 software (LDI, Moore, London, UK) and the area of vasodilatation was defined as those pixels in which intensity exceeded the mean of basal values plus two standard deviations [47].

Differential conduction blockade of myelinated nerve fibers was achieved by placing a padded sling connected to a 2.5 kg weight at the proximal wrist of the volunteers for 30–40 min to apply pressure to the superficial radial nerve (also see [48]). Preferential A-fiber blockade induced by nerve compression has been shown by Torebjork et al. [31] and Mackenzie et al. [32] in microneurography studies. In the case of the superficial radial nerve the impaired A-fiber function with preserved C-fiber function is limited to the autonomous zone of the superficial radial nerve distal the metacarpophalangeal joints I + II. The progress of the differential block following compression of the *N. radialis superficialis* was monitored by decrease of touch and cold detection and was considered to be sufficient when a cold stimulus applied by a metal rod immersed in ice water (7 s, 0°C) was not to be distinguished from a mechanical stimulus. During the whole duration of the block subjects were able to feel pin-prick stimulation as painful sharp pricking, which was interpreted as maintained C-fiber function [49]. When nerve block was established 70 μl of a 100 nM ATX-II containing sterile buffer solution was injected and subjects were asked to rate pain-like and itch-like sensations for 10 min before removal of the block and 10 and 20 min thereafter together with ratings of cold-evoked pain (0°C, 7 s, Figure 7). For the NRS curves of each subject the area under the curve (AUC) was calculated (Figure 7a + b, bar graphs).

### Chemicals

A 5, 25 or 100 nM recombinant ATX-II (Anemonia sulcata toxin, Alomone Labs, Jerusalem, Israel or Sigma-Aldrich, USA) solution was prepared in saline solution (either extracellular recording solution or sterile buffer for injections, which was additionally sterile filtered). TTX (Tetrodotoxin, Biotrend AG, Wangen, Switzerland) was diluted to a final concentration of 500 nM in the extracellular recording solution. For recordings with β4-peptide an internal solution containing 100 μM β4-peptide (KKLITFILKKTREK, PSL GmbH, Heidelberg, Germany, [50]) was prepared and stored on ice during the process of the experiments.

### Statistics

All data are presented as mean ± standard error of the mean (SEM). Paired-sample T-test was used for statistical analysis of DRG paired patch clamp recordings and for N1E115 and DRG activation and steady-state fast inactivation analysis. For temperature effect analysis non-paired Mann Whitney U-test was used for group comparison. For experiments performed on HEK cells and preincubated DRGs independent-sample T-test was used to test for significance. For psychophysics experiments a Wilcoxon matched pairs test was used for intra-individual comparisons and Mann Whitney U-test for the comparison of groups. For RT-qPCR one-tailed Mann Whitney U-test was used for group comparison. Statistical significances are indicated with * for p < 0.05, ** for p < 0.01 and *** for p < 0.001.

## Abbreviations

Nav: Voltage-gated sodium channel; DRGs: Dorsal root ganglion sensory neurons; TTX: Tetrodotoxin; PEPD: Paroxysmal extreme pain disorder; RT-qPCR: Real time quantitative polymerase chain reaction; ATX-II: Sea anemone toxin II; AUC: Area under the curve; CGRP: Calcitonine gene-related peptide; AP: Action potential; ns: Statistically not significant.

## Competing interests

The authors declare that they have no competing interests.

## Authors’ contributions

ABK carried out the patch-clamp recordings and analysis, participated in the acquisition and coordination of psychophysical experiments, carried out FACS sorting data acquisition and interpretation and helped to write the manuscript. ME planned, coordinated and acquired psychophysical studies, analyzed psychophysical data and participated in writing the manuscript. ASL designed, performed and analyzed the RT-qPCR experiments and critically revised the manuscript. BN planned and participated in acquiring and analyzing psychophysical data and critically revised the manuscript. LKK carried out patch-clamp recordings of Nav1.6 and data analysis. ETS contributed to conception and design and carried out patch-clamp recordings and analysis. RS contributed to conception and design of the study, participated in data acquisition and critically revised the manuscript. THo participated in planning the experiments and critically revised the manuscript. CA participated in analyzing the patch-clamp data. THu contributed to conception and design, participated in analyzing the patch-clamp data. RWC contributed to conception and design of the study. AL conceived and designed the patch-clamp recordings, participated in acquisition and coordination of patch-clamp, FACS sorting and psychophysical data and their analysis and interpretation, and wrote the manuscript. All authors read and approved the final manuscript.

## Supplementary Material

Additional file 1 ** Figure S1.** Activation properties of resurgent currents of large diameter DRGs. (a). Resurgent current–voltage curve normalized to peak inward TTXs resurgent currents for large diameter DRGs. (b). Relative resurgent conductance of peak inward TTXs resurgent currents, pre (black squares) and post (pink squares) ATX-II application at 22°C (filled squares, n = 8) and 30°C (open squares n = 14) for large diameter DRGs. Midpoints of activation of relative resurgent conductance were retrieved from a Boltzmann fit and are not significantly different (control 22°C: -57.5 ± 1.4 mV, post ATX-II 22°C: -57.5 ± 1.7 mV, control 30°C: -53.9 ± 1.2 mV, post ATX-II 30°C: -57.7 ± 1.7 mV; Wilcoxon matched pairs test for ATX-II effect: 22°C p = 0.889, 30°C p =0.099; Mann–Whitney-U-test for temperature dependent effect: pre 22°C vs. 30°C p = 0.054, post 22°C vs. 30°C p = 0.758). Click here for file

Additional file 2 **Figure S2.** ATX-II shifts voltage-dependence of activation and steady-state fast inactivation to more hyperpolarized potentials in both large and small DRGs. (a). Voltage-dependence of activation and steady-state fast inactivation of large (left, n = 11–17) and small (right, n = 21-22) DRGs. Vhalf of activation of both cell types is shifted due to exposure to ATX-II significantly (large control: -42.6 ± 1.1 mV, ATX-II: -45.0 ± 0.94 mV, p < 0.01, small control: -34.9 ± 1.0 mV, ATX-II: -40.7 ± 0.8 mV, p < 0.001, paired-sample T-test) whereas slope is not significantly different from control (large control: 2.76 ± 0.22 ATX-II: 2.96 ± 0.23, p = 0.23, small control: 5.66 ± 0.3, ATX-II: 5.7 ± 0.4, p = 0.84, paired-sample T-test). For inactivation, Vhalf of both cell types is shifted significantly (p large < 0.001, p small < 0.001, paired-sample T-test), Vhalf for steady-state inactivation was for large DRGs control: -62.5 ± 1.2 mV, ATX-II: -66.8 ± 1.2 mV, p < 0.001, small DRGs control: - 74.7 ± 2.4 mV, ATX-II: - 80.5 ± 2.0 mV, p < 0.001, paired-sample T-test). (b) and (c): Results from a double exponential fit to current decay of traces evoked by the activation protocol from large and small DRGs. (b). Fractional A1 (A1/(A1 + A2)) is shown as a function of voltage. (c). Fast τ1 and slow τ2 time constants of current decay are shown as a function of voltage. We could not detect any significant changes in the decay time constants of small or large DRGs. Click here for file

Additional file 3 ** Figure S3.** ATX-II fails to evoke resurgent currents in small diameter DRGs also at higher concentrations. (a) Recordings of small DRGs after application of 25 nM ATX-II to the bath solution. Representative resurgent current traces, shown on a magnified time scale on the right. There was no resurgent current detectable although persistent and tail currents are induced by 25 nM ATX-II. (b) TTXs peak current at the time point at which resurgent currents would be expected as a function of voltage. While there is nearly no tail current under control conditions (black squares), application of 25 nM ATX-II increased tail currents, albeit not significantly (pink squares, not significant in a paired-sample T-test, n = 5). (c) Same test setting as shown in (b) but tail currents were excluded in the analysis, leaving only the first part of the slowly declining persistent current detectable (* p < 0.05, paired-sample T-test). (d) Persistent current seems to be affected by application of 25nM ATX-II (pink squares, * p < 0.05, paired-sample T-test, n = 5). Click here for file
