# Supplementary figures and images for: Sea-anemone toxin ATX-II elicits A-fiber-dependent pain and enhances resurgent and persistent sodium currents in large sensory neurons
Source: Mol Pain. 2012 Sep 15;8:69. doi: 10.1186/1744-8069-8-69 (PMC3495684; doi:10.1186/1744-8069-8-69)

Additional Fig 1

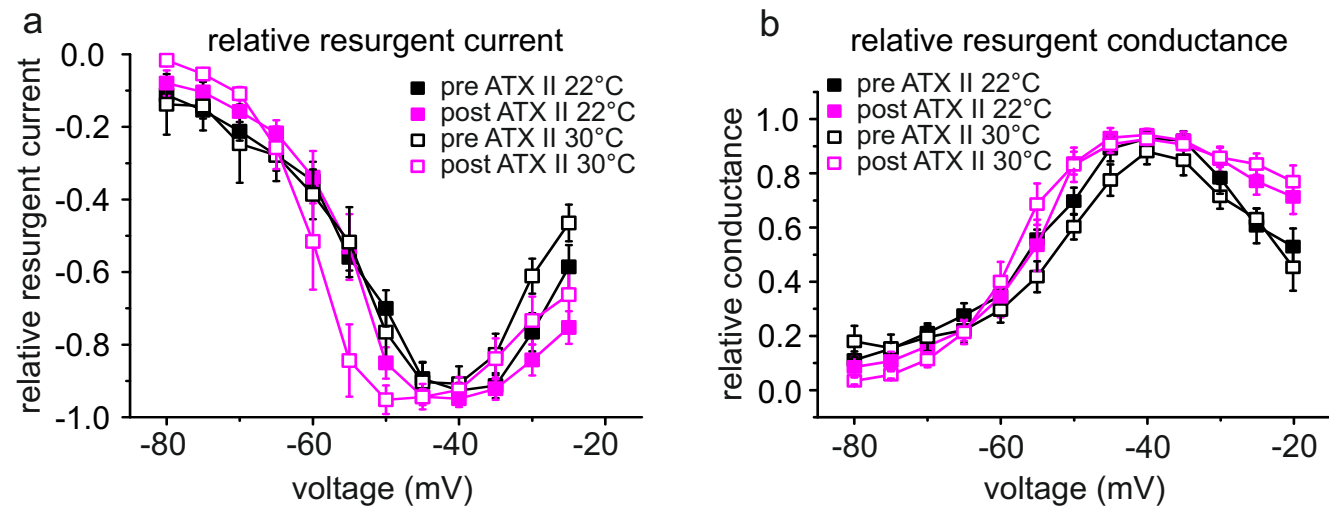

Supplement: Additional file 1 — Figure S1. Activation properties of resurgent currents of large diameter DRGs. (a). Resurgent current–voltage curve normalized to peak inward TTXs resurgent currents for large diameter DRGs. (b). Relative resurgent conductance of peak inward TTXs resurgent currents, pre (black squares) and post (pink squares) ATX-II application at 22°C (filled squares, n = 8) and 30°C (open squares n = 14) for large diameter DRGs. Midpoints of activation of relative resurgent conductance were retrieved from a Boltzmann fit and are not significantly different (control 22°C: -57.5 ± 1.4 mV, post ATX-II 22°C: -57.5 ± 1.7 mV, control 30°C: -53.9 ± 1.2 mV, post ATX-II 30°C: -57.7 ± 1.7 mV; Wilcoxon matched pairs test for ATX-II effect: 22°C p = 0.889, 30°C p =0.099; Mann–Whitney-U-test for temperature dependent effect: pre 22°C vs. 30°C p = 0.054, post 22°C vs. 30°C p = 0.758). [file 1744-8069-8-69-S1.pdf]

Additional Fig 2

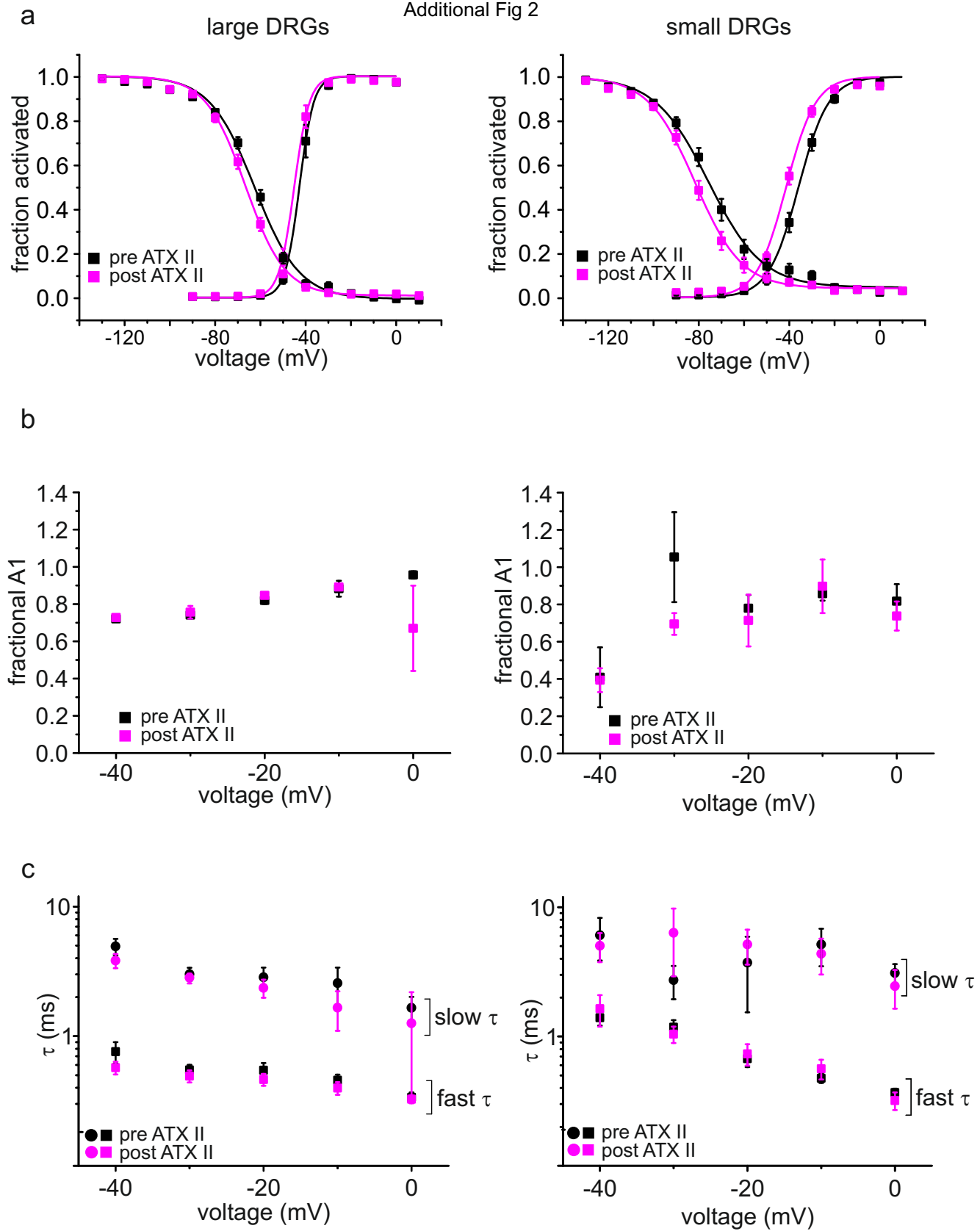

Supplement: Additional file 2 — Figure S2. ATX-II shifts voltage-dependence of activation and steady-state fast inactivation to more hyperpolarized potentials in both large and small DRGs. (a). Voltage-dependence of activation and steady-state fast inactivation of large (left, n = 11–17) and small (right, n = 21-22) DRGs. Vhalf of activation of both cell types is shifted due to exposure to ATX-II significantly (large control: -42.6 ± 1.1 mV, ATX-II: -45.0 ± 0.94 mV, p < 0.01, small control: -34.9 ± 1.0 mV, ATX-II: -40.7 ± 0.8 mV, p < 0.001, paired-sample T-test) whereas slope is not significantly different from control (large control: 2.76 ± 0.22 ATX-II: 2.96 ± 0.23, p = 0.23, small control: 5.66 ± 0.3, ATX-II: 5.7 ± 0.4, p = 0.84, paired-sample T-test). For inactivation, Vhalf of both cell types is shifted significantly (p large < 0.001, p small < 0.001, paired-sample T-test), Vhalf for steady-state inactivation was for large DRGs control: -62.5 ± 1.2 mV, ATX-II: -66.8 ± 1.2 mV, p < 0.001, small DRGs control: - 74.7 ± 2.4 mV, ATX-II: - 80.5 ± 2.0 mV, p < 0.001, paired-sample T-test). (b) and (c): Results from a double exponential fit to current decay of traces evoked by the activation protocol from large and small DRGs. (b). Fractional A1 (A1/(A1 + A2)) is shown as a function of voltage. (c). Fast τ1 and slow τ2 time constants of current decay are shown as a function of voltage. We could not detect any significant changes in the decay time constants of small or large DRGs. [file 1744-8069-8-69-S2.pdf]

Additional Fig 3

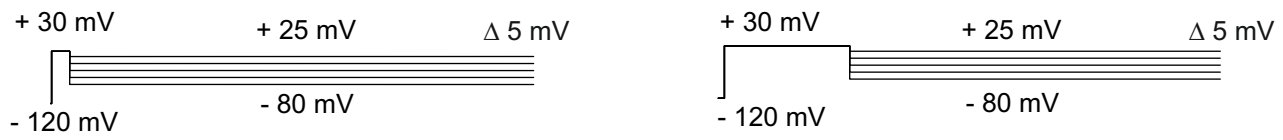

a small DRGs 25 nM ATX

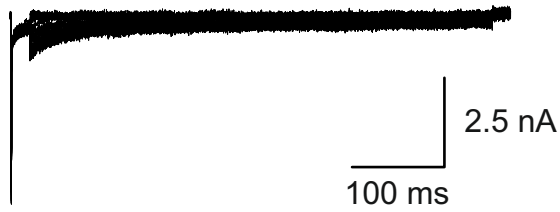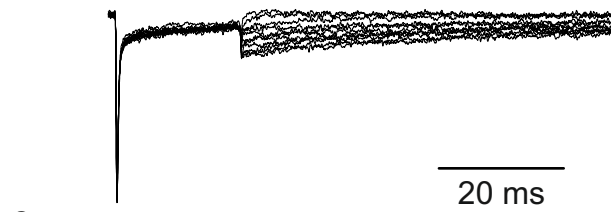

b

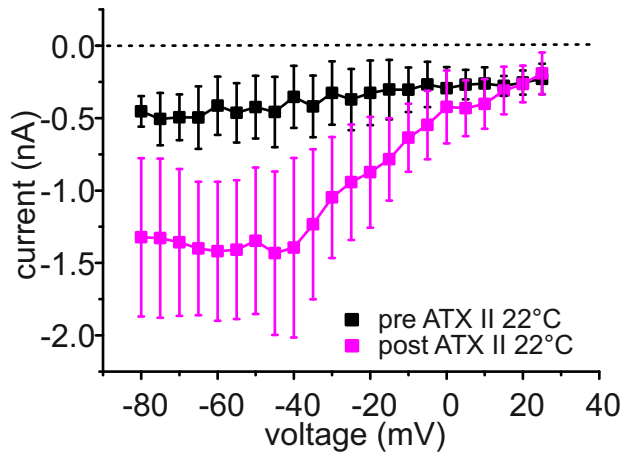

c

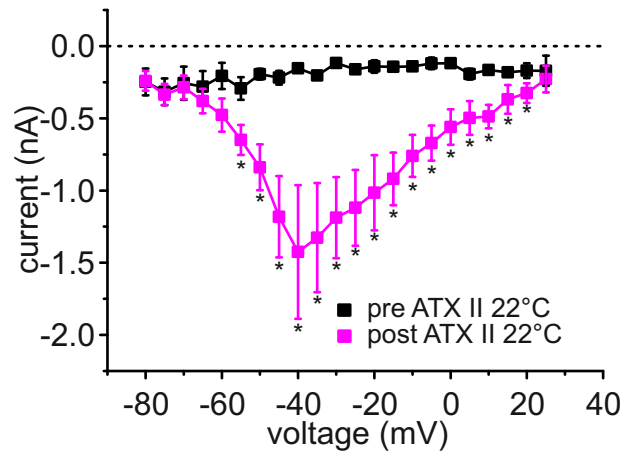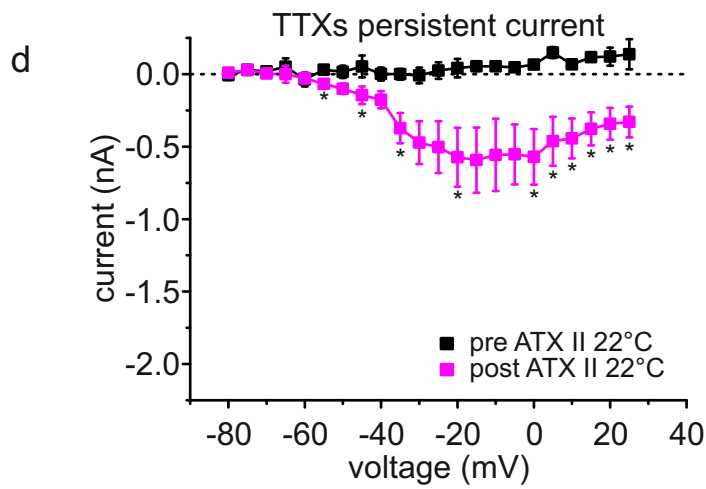

Supplement: Additional file 3 — Figure S3. ATX-II fails to evoke resurgent currents in small diameter DRGs also at higher concentrations. (a) Recordings of small DRGs after application of 25 nM ATX-II to the bath solution. Representative resurgent current traces, shown on a magnified time scale on the right. There was no resurgent current detectable although persistent and tail currents are induced by 25 nM ATX-II. (b) TTXs peak current at the time point at which resurgent currents would be expected as a function of voltage. While there is nearly no tail current under control conditions (black squares), application of 25 nM ATX-II increased tail currents, albeit not significantly (pink squares, not significant in a paired-sample T-test, n = 5). (c) Same test setting as shown in (b) but tail currents were excluded in the analysis, leaving only the first part of the slowly declining persistent current detectable (* p < 0.05, paired-sample T-test). (d) Persistent current seems to be affected by application of 25nM ATX-II (pink squares, * p < 0.05, paired-sample T-test, n = 5). [file 1744-8069-8-69-S3.pdf]
